# The Emerging Role of Glucagon-like Peptide-1 Receptor Agonists in the Management of Obesity-Related Heart Failure with Preserved Ejection Fraction: Benefits beyond What Scales Can Measure?

**DOI:** 10.3390/biomedicines12092112

**Published:** 2024-09-16

**Authors:** Paschalis Karakasis, Nikolaos Fragakis, Dimitrios Patoulias, Panagiotis Theofilis, Marios Sagris, Theocharis Koufakis, Panayotis K. Vlachakis, Imran Rashid Rangraze, Mohamed El Tanani, Konstantinos Tsioufis, Manfredi Rizzo

**Affiliations:** 1 Second Department of Cardiology, Hippokration General Hospital, Aristotle University of Thessaloniki, 54642 Thessaloniki, Greece; fragakis.nikos@googlemail.com; 2 Second Propedeutic Department of Internal Medicine, Faculty of Medicine, School of Health Sciences Aristotle, University of Thessaloniki, 54642 Thessaloniki, Greece; dipatoulias@gmail.com (D.P.); thkoyfak@hotmail.com (T.K.); 3 First Cardiology Department, School of Medicine, Hippokration General Hospital, National and Kapodistrian University of Athens, 11528 Athens, Greece; panos.theofilis@hotmail.com (P.T.); masagris1919@gmail.com (M.S.); vlachakispanag@gmail.com (P.K.V.); ktsioufis@gmail.com (K.T.); 4 Ras Al Khaimah Medical and Health Sciences University, Ras Al Khaimah P.O. Box 11172, United Arab Emirates; imranrashid@rakmhsu.ac.ae (I.R.R.); eltanani@rakmhsu.ac.ae (M.E.T.); manfredi.rizzo@unipa.it (M.R.); 5 School of Medicine, Department of Health Promotion, Mother and Child Care, Internal Medicine and Medical Specialties (Promise), University of Palermo, 90100 Palermo, Italy

**Keywords:** heart failure with preserved ejection fraction, HFpEF, obesity, glucagon-like peptide-1 receptor agonists

## Abstract

Obesity is a significant predisposing factor for heart failure with preserved ejection fraction (HFpEF). Although a substantial proportion of individuals with HFpEF also have obesity, those with obesity are under-represented in clinical trials for heart failure. In turn, current guidelines provided limited recommendations for the medical management of this patient population. Both obesity and diabetes induce a pro-inflammatory state that can contribute to endothelial dysfunction and coronary microvascular impairment, finally resulting in HFpEF. Additionally, obesity leads to increased epicardial and chest wall adiposity, which enhances ventricular interdependence. This condition is further aggravated by plasma and blood volume expansion and excessive vasoconstriction, ultimately worsening HFpEF. Despite the well-documented benefits of GLP-1 receptor agonists in subjects with diabetes, obesity, or both, their role in obesity-related HFpEF remains unclear. In light of the recently published literature, this review aims to investigate the potential mechanisms and synthesize the available clinical evidence regarding the role of GLP-1 receptor agonists in patients with obesity-related HFpEF.

## 1. Introduction

Heart failure (HF) represents a significant global public health challenge, with an estimated prevalence exceeding 64 million individuals worldwide [1]. In the United States, more than 6 million adults are affected by HF, with projections indicating this number could rise to 8 million by 2030 [2]. Similar trends are observed on a global scale [3]. Notably, over half of those diagnosed with HF have preserved ejection fraction (HFpEF), and its prevalence is increasing in comparison to HF with reduced ejection fraction [4,5]. The growing incidence of HFpEF is associated with factors such as an aging population, and increasing prevalence of hypertension, metabolic syndrome, diabetes, and atrial fibrillation—comorbidities that are closely linked to the accumulation of excess body fat [6].

**Figure 1:**
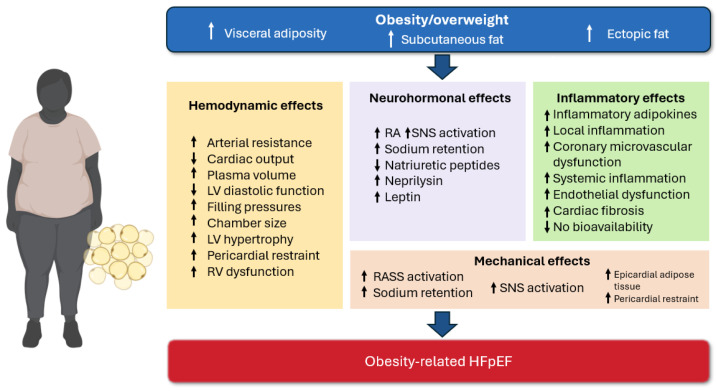
The pathophysiological mechanisms underlying obesity-related alterations in cardiac structure and function involve a complex interplay of inflammatory processes and cardiovascular remodeling. Obesity triggers a transformation of adipose tissue into a pro-inflammatory state, which can adversely affect the vasculature and various visceral organs. In heart failure with preserved ejection fraction (HFpEF) patients, coronary microvascular endothelial dysfunction is evidenced by elevated expression of endothelial adhesion molecules, such as vascular cell adhesion molecule (VCAM) and E-selectin, observed in myocardial biopsy specimens. Additionally, pro-inflammatory cytokines stimulate the endothelial generation of reactive oxygen species (ROS) through the activation of nicotinamide adenine dinucleotide phosphate (NADPH) oxidases, leading to increased nitrosative and oxidative stress within the myocardium of HFpEF patients. Obesity, along with increased visceral adiposity, is further associated with significant abnormalities in cardiac structure and function, including increased left ventricular (LV) mass, enhanced LV concentric hypertrophy, and more severe LV diastolic dysfunction. The presence of excess adipose tissue also correlates with plasma volume expansion and impaired LV relaxation, likely driven by systemic inflammation. These pathophysiological changes may contribute to reduced ventricular compliance, elevated LV filling pressures, and the characteristic clinical manifestations of HFpEF. Abbreviations: LV, left ventricular; RV, right ventricular; RAAS, renin-angiotensin-aldosterone system; SNS, sympathetic nervous system; NO, nitric oxide; HFpEF, heart failure with preserved ejection fraction.

Since 1980, the prevalence of obesity has more than doubled in over 70 countries [7]. In the United States, the rate of obesity now surpasses 40%, a dramatic rise from just 15% in the 1970s [8]. Projections indicate that by 2030, half of the adults in the U.S. will be classified as obese [8]. Epidemiological research has established a robust and independent association between obesity and the development of HFpEF **(**Figure 1**)** [9,10,11,12]. Unlike atherosclerotic vascular diseases, the association between obesity and HF cannot be fully accounted for by traditional cardiovascular risk factors [13]. Despite growing awareness, HFpEF remains frequently overlooked as a cardiovascular complication in individuals with excess body fat, leading to both underdiagnosis and insufficient treatment of obesity-related HFpEF [14].

Despite extensive epidemiological evidence reinforcing the link between obesity and HFpEF, the inclusion of obese HFpEF patients in clinical trials remains notably restricted [15]. These patients are frequently excluded from studies, either directly due to stringent body mass index (BMI) eligibility criteria or indirectly because of the lower plasma levels of natriuretic peptides, which are intrinsically affected by obesity [16,17]. Taken together with the fact that HFpEF is a multifaceted syndrome with multiple subphenotypes [18], each with a distinct pathophysiological background, current guideline recommendations for managing HFpEF remain less clear beyond the use of sodium–glucose cotransporter 2 (SGLT2) inhibitors [19,20,21,22,23] and diuretics [24,25].

Over the past decade, glucagon-like peptide-1 receptor agonists (GLP-1 RAs) have emerged as highly effective glucose and body-weight-lowering agents for individuals with type 2 diabetes (T2DM), obesity, or both [26,27,28,29,30,31,32,33]. T2DM is prevalent among patients with HFpEF and is associated with adverse hemodynamic and clinical characteristics, including increased symptom burden and reduced functional capacity compared to patients without T2DM [34]. Additionally, individuals with obesity-related HFpEF often experience more severe symptoms, impaired functional status, decreased quality of life, and poorer clinical outcomes compared to those without obesity [35,36,37]. Given the well-established therapeutic role of GLP-1 RAs in managing T2DM and/or overweight/obesity, and their close interrelationship with HFpEF, scientific focus has shifted to investigating the role of GLP-1 RAs in obesity-related HFpEF, regardless of T2DM status. As highlighted by a recent international consensus, a modern holistic approach that manages the cardiometabolic continuum—from overweight and obesity to prediabetes and type 2 diabetes—can lead to longer and healthier lives for patients and help in preventing related complications [38]. Despite the known benefits of GLP-1RAs in managing diabetes and obesity, their role in HFpEF, particularly in relation to obesity, remains underexplored. Therefore, the present review aims to investigate the potential mechanisms and consolidate the existing clinical evidence regarding the emerging role of GLP-1 RAs in individuals with obesity-related HFpEF.

## 2. Potential Mechanisms Underlying the Effects of GLP-1RAs on Obesity-Related HFpEF

**Figure 2:**
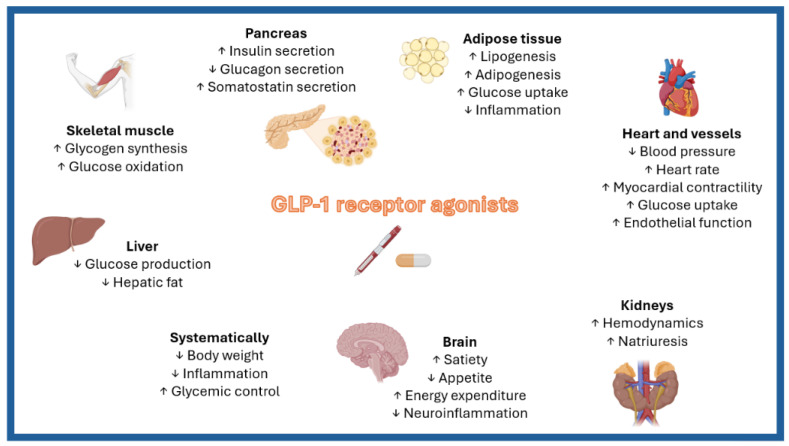
The pleiotropic effects of glucagon-like peptide-1 receptor agonists (GLP-1 RAs) and their potential role in management of obesity-related heart failure with preserved ejection fraction (HFpEF).

Similar to many chronic diseases, obesity paradoxically offers protection against adverse outcomes in patients with established HFpEF, even though it is a well-known predisposing factor for the development of this condition (Table 1). However, these findings are primarily derived from observational studies, which are inherently limited by survival bias and the potential for reverse causation. Conversely, evidence from Mendelian randomization analyses indicates that each 1 kg/m^2^ increase in BMI is associated with a significant 19% increase in the incidence of HF and a 27% increase in HF-related mortality [39]. Despite the ongoing debate surrounding the obesity paradox, GLP-1RAs are hypothesized to confer benefits to individuals with cardiometabolic HFpEF through their remarkable pleiotropic effects (Figure 2).

### 2.1. GLP-1RAs and Epicardial Adipose Tissue

The accumulation of epicardial adipose tissue (EAT) is an essential driver of systemic inflammation in populations with different ethnicities [48] and is linked to increased production of pro-inflammatory cytokines (leptin, tumor necrosis factor-alpha, interleukin (IL)-1B and IL-6, and resistin), myocardial fibrosis (through activin A, visfatin, transforming growth factor-β1, and monocyte chemoattractant protein-1, which have been linked to fibroblast proliferation), ventricular hypertrophy, and elevated cardiac filling pressures, all of which are characteristic features of HFpEF [49,50]. Moreover, EAT is associated with pericardial restraint, enhanced ventricular interdependence, and uncoupling between the left ventricular (LV) filling pressure and LV preload, pathophysiological changes observed in the unique obese phenotype of HFpEF [51]. Of note, several GLP-1RAs, including semaglutide, liraglutide, dulaglutide, and exenatide, have demonstrated efficacy in remodeling epicardial adipose tissue, resulting in significant dose-dependent reductions in EAT thickness ranging from 13% to 42% (Table 2).

### 2.2. GLP-1RAs, Renal Function, and Diuretic Resistance

GLP-1RAs and other incretin-based therapies may exert several potential decongestive effects in obesity-related HFpEF. Firstly, GLP-1RA treatment has been shown to improve short-term renal arteriolar vasodilation, enhance renal blood flow, and promote natriuresis, potentially through mechanisms involving the GLP-1 receptor, inhibition of the Na+/H+ exchanger 3, and reduced neurohormonal activation—effects that may be particularly pronounced in the context of circulatory congestion [56,57]. Additionally, these mechanisms could mitigate reflex renal vasoconstriction induced by loop diuretics, a significant contributor to diuretic resistance. Furthermore, GLP-1RAs have demonstrated reductions in renal inflammation and preservation of renal structure and function, as evidenced by findings from the recent FLOW trial [58]. Beneficial kidney effects of GLP-1RAs, including reduced tubulointerstitial damage, and glomerulosclerosis, along with improved podocyte architecture, have already been described in preclinical studies [59]; however, the mechanisms underlying these findings remain speculative and—undoubtedly—warrant further investigation. The final clinical result of those beneficial effects, that of significant reduction in albuminuria levels and conservation of renal function, is of utmost importance for the management of patients with obesity-related HFpEF [60], based on the significant prognostic role of albuminuria in this population in terms of adverse cardiovascular outcomes.

### 2.3. GLP-1RAs and the Renin–Angiotensin System

GLP-1 receptor activation has been shown to potentially interact with the renin–angiotensin system. Skov et al. were the first to report that GLP-1 infusion reduced angiotensin II levels and promoted natriuresis in healthy young men [61]. Additionally, Le et al. demonstrated that the activation of the GLP-1 receptor by exendin-4 decreased the intrarenal renin–angiotensin system activity in mice, which led to reduced angiotensin II-mediated signaling through the transforming growth factor-beta 1/SMAD family member 3 pathway [62]. Indeed, clinical data have suggested that the effects of GLP-1 RAs on the renin–angiotensin system might mediate the beneficial cardiovascular effects of this class in individuals with T2DM [63].

### 2.4. GLP-1RAs and Microvascular Dysfunction

Coronary microvascular endothelial dysfunction in HFpEF is characterized by an elevated expression of endothelial adhesion molecules, including vascular cell adhesion molecule (VCAM) and E-selectin, as evidenced in myocardial biopsy specimens [64,65]. Additionally, pro-inflammatory cytokines stimulate the production of reactive oxygen species by the endothelium via the activation of nicotinamide adenine dinucleotide phosphate oxidases [66]. This contributes to significant nitrosative and oxidative stress, promoting a status of cardiac hypoxia/pseudohypoxia, which is prevalent in the myocardium of HFpEF [67] and is further exacerbated by common comorbidities, like type 2 diabetes mellitus and age-related physiological changes [68].

In a substudy of the LIRAFLAME trial, a randomized, double-blind, placebo-controlled investigation into the impact of liraglutide on the plasma lipidome, 30 participants were randomly assigned to receive either liraglutide or placebo [69]. These participants underwent hybrid [64Cu]-DOTATATE positron emission tomography and computed tomography of the heart both at baseline and following 26 weeks of treatment. Notably, coronary artery inflammation, measured as changes in SUVmax and mSUVmax, significantly declined in the liraglutide group but not in the placebo group [69]. Moreover, preclinical studies suggest that GLP-1RAs exert beneficial effects on coronary microvascular function. For instance, a study investigating the impact of liraglutide on lean and obese rats on a high-salt diet observed an improved dilatory response of microvessels to acetylcholine [70]. Additionally, research conducted on diabetic rats demonstrated that exenatide preserved cardiac microvascular integrity and reduced the diffusion of lanthanum nitrate across endothelial cells, highlighting its protective effects on cardiac microvascular barrier function [71].

### 2.5. GLP-1RAs and Atrial Fibrillation

Atrial fibrillation (AF) is common in patients with HFpEF, with prevalence rates reported to be as high as 65% [72,73,74]. Patients with AF have a more unfavorable prognosis than those in sinus rhythm, with the elevated risk associated with AF being more significant in HFpEF than in HFrEF [75]. Evidence from the DELIVER trial, which enrolled 6263 patients with HFpEF, demonstrated a significantly higher risk of HF hospitalization across all AF subtypes compared to AF-free participants [76]. Moreover, in the context of HFpEF, obesity-mediated structural and electrical remodeling further contributes to AF progression and is linked to higher rates of incident AF [77].

A recent meta-analysis identified a strong correlation between elevated glycemic variability and an increased risk of developing AF [78]. Among antidiabetic therapies, GLP-1RAs have been demonstrated to be the most effective in mitigating glycemic variability [79]. Additionally, prior research has indicated that GLP-1 receptors are abundantly expressed in the atrial myocardium, implying a potential role for GLP-1 in the regulation of atrial function [80]. These findings, which establish a pathophysiological context potentially limiting the benefits of GLP-1RAs in AF management, were substantiated by a network meta-analysis conducted by Shi et al. [81]. This analysis, which included data from the general population, revealed that GLP-1RAs were significantly associated with a reduced incidence of AF in diabetic patients when compared to other glucose-lowering medications [81]. Comparable results have been recently reported with GLP-1RAs in the setting of AF ablation [82,83]. Collectively, this evidence underscores the powerful role of this novel drug class in managing AF, tackling its devastating effects in patients with obesity-related HFpEF.

### 2.6. GLP-1RAs and Myocardial Energetics

**Figure 3:**
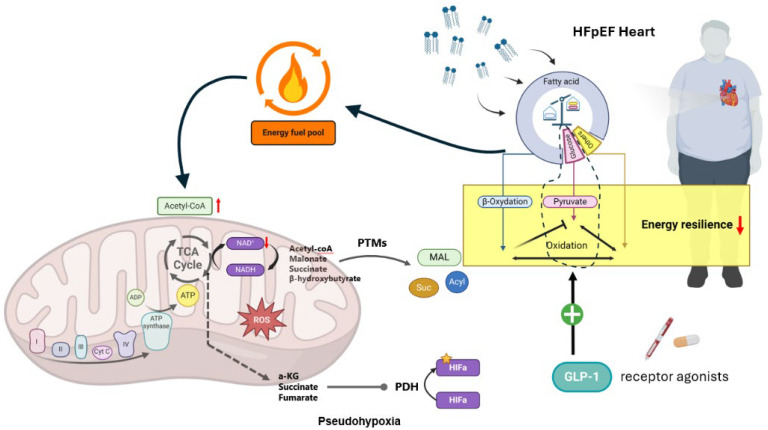
In the context of cardiometabolic heart failure with preserved ejection fraction (HFpEF), significant energetic alterations occur. Under normal physiological conditions, the heart primarily relies on fatty acids (approximately 70%) for adenosine triphosphate (ATP) production, supplemented by glucose, branched-chain amino acids, ketone bodies, and lactate. The healthy heart exhibits metabolic flexibility, enabling it to switch between these energy substrates based on nutrient availability. However, in the setting of obesity-associated HFpEF, an excess influx of fatty acids disturbs the balance among these energy substrates, detrimentally affecting cardiac energy homeostasis—for instance, by inhibiting glucose oxidation (as illustrated in the right portion of the figure). This disruption in the physiological substrate oxidation pattern leads to an imbalance in the redox state and alters metabolite availability, which, in turn, modulates cellular signaling pathways through post-translational modifications (PTMs). This imbalance also increases oxidative and nitrosative stress (e.g., reactive nitrogen/oxygen species (R/NOS) production), fostering a state of cardiac hypoxia or pseudohypoxia. Glucagon-like peptide-1 receptor agonists (GLP-1RAs) have been shown to favor glucose oxidation, thereby restoring metabolic flexibility and providing protection against cardiac pseudohypoxia.Abbreviations: ATP, adenosine triphosphate; HIF, hypoxia-inducible factor; GLP-1, glucagon-like peptide-1; NAD^+^/NADH, Nicotinamide-adenine dinucleotide PDH, pyruvate dehydrogenase; PTMs, post-translational modifications; TCA, tricarboxylic acid; ROS, reactive oxygen species.

In obese HFpEF, the excessive influx of fatty acids (FAs) forces the heart to maintain or even increase its dependence on FA oxidation, deviating from the expected metabolic shift towards glucose oxidation observed in HF with reduced ejection fraction (HFrEF) [84]. To capture the complexity of the metabolic alterations in cardiometabolic HFpEF, where the heart is likely characterized by an energy deficit, the concept of ‘energy resilience’ has recently been introduced. This term reflects the heart’s altered metabolic state in HFpEF, which appears predisposed to preferentially utilize the most abundant substrate, in this case, FAs. However, prolonged reliance on FA oxidation can lead to detrimental outcomes, including lipid accumulation and subsequent cardiac lipotoxicity, which contribute to myocardial maladaptation in HFpEF. Therefore, reestablishing the balance between energy substrates—referred to as metabolic resilience—is essential for enabling the heart to adapt its energy production effectively in response to fluctuating energy demands [85,86]. Of note, GLP-1RAs have been demonstrated to improve myocardial glucose oxidation [87,88]. Consequently, GLP-1RAs may play a pivotal role in reestablishing energy resilience and metabolic flexibility, thereby alleviating the adverse effects associated with the previously outlined pathophysiological mechanisms (Figure 3) [89].

## 3. GLP-1RAs and Clinical Outcomes in Obesity-Related HFpEF

Even though GLP-1RAs have been shown to marginally reduce the risk of hospital admission for HF in patients with T2DM [90], their role across various HF phenotypes, especially in those without T2DM, remains insufficiently explored. Similar to SGLT2 inhibitors, although GLP-1RAs were initially designed for glycemic management in patients with type 2 diabetes, their impressive effects beyond glucose control, particularly in reducing cardiovascular risk and promoting weight loss, have prompted consideration of their potential use in patients without concurrent T2DM [31]. In late 2023, the SELECT trial (Semaglutide Effects on Cardiovascular Outcomes in People with Overweight or Obesity) assessed 17,604 patients with pre-established cardiovascular disease who were overweight or obese but did not have diabetes [91]. Over a 5-year period, weekly subcutaneous administration of semaglutide reduced the risk of cardiovascular death, nonfatal myocardial infarction, or nonfatal stroke by 20%. Additionally, one of the trial’s secondary endpoints, which focused on a composite HF outcome (including cardiovascular death, hospitalization, or urgent medical visits for HF), showed an 18% reduction by the trial’s end [91]. Although preliminary, these findings underscored the potential of GLP-1RAs in preventing HF in patients with obesity and cardiovascular disease, even in the absence of diabetes, and further highlighted the clinical relevance of semaglutide in overweight/obese patients for atherosclerotic cardiovascular disease prevention [92].

The STEP-HFpEF program recently provided novel evidence on the emerging role of GLP-1RAs in obesity-related HFpEF through HF-dedicated randomized controlled trials (RCTs) and their prespecified secondary analyses (Table 3) [93].

### 3.1. GLP-1RAs and Clinical Outcomes in Obesity-Related HFpEF in Patients without Diabetes

The STEP-HFpEF trial enrolled 529 patients with HFpEF and a BMI of 30 or above, assigning them to either once-weekly semaglutide (2.4 mg) or placebo over a 52-week period. The coprimary outcomes were the change from baseline in the Kansas City Cardiomyopathy Questionnaire clinical summary score (KCCQ-CSS), where higher scores reflect more mild symptoms and physical limitations, and the change in body weight. Of note, semaglutide led to a substantial increase in the KCCQ-CSS, with a rise of 16.6 points compared to 8.7 points with placebo, yielding an estimated significant difference of 7.8 points. Additionally, semaglutide significantly reduced body weight by 13.3% compared to a 2.6% reduction with placebo, resulting in an estimated significant difference of −10.7 percentage points. Considering the secondary confirmatory endpoints, semaglutide significantly improved the 6 min walk distance (6MWD) by 21.5 m compared to 1.2 m with placebo. In the hierarchical composite endpoint, which included mortality, HF events, and changes in the KCCQ-CSS and 6MWD, semaglutide outperformed placebo with a win ratio of 1.72 (95% CI, 1.37–2.15; *p* < 0.001). The drug also markedly reduced C-reactive protein (CRP) levels by 43.5% compared to a 7.3% reduction with placebo. Fewer serious adverse events were observed in the semaglutide arm (13.3%) compared to the placebo (26.7%).

Butler et al. presented a prespecified analysis of the STEP-HFpEF trial stratifying patients according to baseline left ventricular ejection fraction (LVEF, 45% to 49%, 50% to 59%, and ≥ 60%) [95]. Semaglutide significantly enhanced symptoms, alleviated physical limitations, improved exercise capacity, and decreased inflammation and body weight consistently across all LVEF categories, implying that it is beneficial for individuals with the obesity phenotype of HFpEF, irrespective of their LVEF [95]. Interestingly, another subanalysis indicated improvements in HF-related symptoms, physical limitations, exercise function, inflammation, body weight, and NT-proBNP levels, regardless of baseline health status as assessed by KCCQ-CSS tertiles [96]. Lastly, Borlaug et al. demonstrated that the benefits of semaglutide were consistent across obesity classes I to III (BMI 30.0–34.9 kg/m^2^, 35.0–39.9 kg/m^2^, and ≥ 40 kg/m^2^), with the degree of benefit being directly proportional to the extent of weight loss achieved [97].

### 3.2. GLP-1RAs and Clinical Outcomes in Obesity-Related HFpEF in Patients with Diabetes

The STEP-HFpEF DM trial, published earlier this year, randomized 616 patients with the same inclusion criteria as the STEP-HFpEF trial but with the addition of T2DM at baseline, suggesting that semaglutide’s benefits also extend to individuals with obesity-related HFpEF and coexisting T2DM [94]. Briefly, semaglutide led to a mean increase of 13.7 points in the KCCQ-CSS compared to 6.4 points with placebo, yielding an estimated difference of 7.3 units (95% CI 4.1–10.4; *p* < 0.001) [94]. Additionally, semaglutide resulted in a 9.8% reduction in body weight compared to a 3.4% reduction with placebo, with an estimated between-group difference of 6.4 percentage units (95% CI, 7.6 to 5.2; *p* < 0.001). Confirmatory secondary endpoints also favored semaglutide, with improvements in 6MWD (14.3 m; 95% CI 3.7–24.9; *p* = 0.008), a higher win ratio for the hierarchical composite endpoint (1.58; 95% CI 1.29–1.94; *p* < 0.001), and a greater reduction in CRP levels (estimated treatment ratio, 0.67; 95% CI 0.55–0.80; *p* < 0.001) [94]. Serious adverse events were reported in 17.7% of the semaglutide arm compared to 28.8% in the placebo arm [94].

### 3.3. GLP-1RAs and Clinical Outcomes in Obesity-Related HFpEF Irrespective of T2DM Status

In view of the relatively modest size of both trials, Butler et al. presented a combined analysis of STEP-HFpEF and STEP-HFpEF-DM comprising a total of 1145 individuals with an obesity phenotype of HFpEF with or without T2DM [98]. Collectively, improvements in the KCCQ-CSS and reductions in body weight over 52 weeks were significantly greater in the semaglutide arm compared to the placebo arm, with a mean difference of 7.5 points in the KCCQ-CSS and 8.4% in body weight. For confirmatory secondary endpoints, semaglutide significantly improved 6MWD (mean difference 17.1 m; 95% CI 9.2 to 25.0) and the hierarchical composite endpoint (win ratio 1.65; 95% CI 1.42 to 1.91), while also significantly reducing CRP levels [98]. The efficacy of semaglutide on the dual primary endpoints was largely consistent across subgroups defined by age, race, BMI, systolic blood pressure, baseline CRP, and LVEF [98]. Regarding safety endpoints, the trialists emphasized that semaglutide was well tolerated, with 161 serious adverse events reported in the semaglutide group compared to 301 in the placebo group [98]. Importantly, two subgroup analyses based on NYHA functional class and NT-proBNP levels demonstrated that these benefits were consistent across all NYHA functional class categories, with particularly pronounced improvements in health status observed in patients with NYHA classes III/IV and higher baseline NT-proBNP, suggesting greater benefits for participants with more severe disease status [99].

#### 3.3.1. Effect of GLP-1RAs According to Baseline Diuretic Use and Diuretic Resistance

Patients with HFpEF often receive loop diuretics, which, while essential for decongestion, can lead to electrolyte imbalances, deteriorating renal function, and hypotension [104]. Additionally, increases in outpatient loop diuretic doses are linked with adverse outcomes and are increasingly seen as indicators of HF hospitalizations [94,105,106,107]. In HFpEF, elevated body mass index correlates with increased use and higher doses of loop diuretics [108,109]. In cases of obesity-related HFpEF, loop diuretics tend to be less effective for decongestion and have a more pronounced negative impact on renal function compared to patients with HFpEF without obesity, highlighting the need for alternative decongestive therapies [110].

Shah et al. leveraging pooled data from the STEP-HFpEF and STEP-HFpEF-DM trials reported that semaglutide consistently delivered positive outcomes in body weight, exercise capacity, and markers of inflammation and congestion across various subgroups based on background diuretic therapy and dosage [101]. It was also well tolerated, showing fewer serious adverse events and cardiac disorders compared to placebo, regardless of baseline diuretic use or dosage. Over the 52-week treatment period, semaglutide resulted in (i) nearly a 20% reduction in the total daily dose of loop diuretics; (ii) more than a two-fold increase in the likelihood of reducing loop diuretic dosage; and (iii) a 66% reduction in the likelihood of increasing the loop diuretic dose [101]. Additionally, semaglutide was associated with fewer new prescriptions for loop diuretics in diuretic-naïve patients at baseline and a higher frequency of discontinuation among diuretic users compared to placebo. Of note, in the semaglutide group, reductions in the daily dose of loop diuretics were linearly related to improvements in health status, decreases in body weight, and reductions in systemic inflammation [101]. Taken together, these findings underscore semaglutide’s effectiveness across the entire spectrum of patients with obesity-related HFpEF, including those who do not require loop diuretics and those with significant congestion necessitating high-dose loop diuretic therapy, often alongside mineralocorticoid receptor antagonists (MRAs) and SGLT2 inhibitors. Despite more modest effects observed with sacubitril/valsartan and SGLT2 inhibitors on diuretic therapy in other HFpEF trials [111,112], which have questioned the need for preemptive loop diuretic dose reductions, the notable rate of loop diuretic de-escalation observed in the STEP-HFpEF program (approximately 1 in 7 participants) indicates that clinicians should consider the potential need for adjusting diuretic doses following the initiation of semaglutide therapy. Of course, this has also to be assessed in line with the potential gastrointestinal adverse events regularly seen within the first weeks after initiation of a GLP-1RA; thus, concomitant use of loop diuretics might also increase the risk of volume depletion phenomena and result in related complications, such as acute kidney injury.

#### 3.3.2. Efficacy of GLP-1RAs by Sex in Obesity-Related HFpEF

Sex exerts a profound influence on various aspects of HF, including risk factors, clinical presentation, treatment efficacy, and prognosis [113]. Notably, HFpEF predominantly affects women, who often endure a greater burden of comorbidities, more severe symptoms, and greater physical limitations compared to men [114,115]. Despite these challenges, women with HFpEF generally experience better survival rates and fewer HF-related hospitalizations. Women with HFpEF are characterized by smaller LV cavity sizes and distinct body composition and adipose distribution compared to men, which may influence the effectiveness of therapies targeting excess body fat [37]. Additionally, the relationship between blood and plasma volume increase with rising body weight is steeper in women than in men [116]. Sex differences also extend to treatment responses. For instance, the PARAGON-HF trial demonstrated a more favorable effect of sacubitril/valsartan on the primary endpoint of total hospitalizations for HF and cardiovascular death in women compared to men with HFpEF [117]. This distinction is particularly significant given that obesity is a more potent risk factor for HFpEF in women than in men and that women with obesity often experience greater weight loss with antiobesity agents than their male counterparts [118,119,120].

Conversely, prior studies have indicated that women with obesity-related HFpEF have significantly higher levels of visceral adiposity, which can negatively affect exercise hemodynamics and exacerbate symptoms more than in men. Visceral adiposity is believed to act as a reservoir for the release of various adipocytokines and neurohormones, contributing to local and systemic inflammation, sodium retention, and plasma volume expansion [35,116]. This elevated symptom burden in women may be attributed to a more pronounced increase in exercise pulmonary capillary wedge pressure due to higher plasma and blood volumes compared to men [116].

Recently, Verma et al. presented new findings from the STEP-HFpEF program, analyzing data from 1145 patients with obesity-related HFpEF, nearly half of whom (49.7%) were women [59]. The study revealed that semaglutide significantly improved the KCCQ-CSS in both women and men, with similar mean differences (women: +7.6 points [95% CI: 4.5–10.7]; men: +7.5 points [95% CI: 4.3–10.6]; P for interaction = 0.94) [59]. Consistent with prior investigations, the reduction in body weight was more pronounced in women compared to men (P for interaction = 0.006). Furthermore, semaglutide led to improvements in the 6MWD and the hierarchical composite endpoint without significant sex-specific interaction [59]. Lastly, a noteworthy observation is that despite greater weight loss in women, this did not translate into proportionate improvements in the clinical and cardiometabolic parameters under investigation compared to male participants. This is in line with recent evidence showing that the weight response to GLP-1RAs is greater in women than in men [118].

## 4. Conclusions

The growing pandemics of T2DM and overweight/obesity have led to a rapid increase in the number of patients with obesity-related HFpEF. However, current evidence on managing this population remains scarce due to their exclusion from landmark heart failure trials. This highlights, firstly, the urgent need to broaden inclusion criteria in future studies to better address this specific HFpEF phenotype, and secondly, the need for novel therapeutic options. Despite the recent introduction of SGLT2 inhibitors into the treatment algorithms for HFpEF, therapeutic options remain exceptionally limited, leaving these patients with increased mortality rates and a diminished quality of life. As we enter the era of personalized medicine, and particularly given the multifaceted nature of HFpEF, the one-size-fits-all approach—reminiscent of Procrustes in Greek mythology—is becoming obsolete. Recently published research provided promising evidence on the efficacy and safety of semaglutide in treating obesity-related HFpEF, regardless of baseline T2DM. This extends to other GLP-1-based agents, such as tirzepatide, a novel dual GLP-1 and glucose-dependent insulinotropic polypeptide (GIP) receptor agonist. The preliminary results from the phase 3 SUMMIT trial showed that compared to placebo, tirzepatide resulted in a significant 38% reduction in the risk of a composite HF endpoint, including urgent visits or hospitalization for HF, oral diuretic intensification, or cardiovascular death. The present review builds upon emerging clinical evidence suggesting that GLP-1RAs may confer benefits beyond weight reduction by modulating significant risk factors and addressing major drivers of obesity-related HFpEF. These include reductions in EAT, improvements in renal function and diuretic resistance, enhancement of microvascular dysfunction and myocardial energetics, modulation of the renin–angiotensin system, and better management of the AF-HFpEF vicious cycle. Thus, the future appears increasingly bright for patients with obesity-related HFpEF, with the introduction and adoption of novel, disease-modifying GLP-1RAs, despite certain limitations, such as high costs and supply shortages observed so far.

Therefore, the therapeutic landscape appears to be constantly changing. Future trials are eagerly anticipated to explore the therapeutic potential of other commercially available GLP-1RAs in cardiometabolic HFpEF and to investigate their impact on the long-term prognosis of this patient population.

## Figures and Tables

**Table 1:** Studies investigating the association between obesity paradox and HFpEF.

Study/Author	Study Design	Sample Size	LVEF	Follow-up Duration	Main Findings
I-PRESERVE, 2011 [40]	RCT	4109	≥50%	49.5 months	After adjusting for 21 risk factors, including age, sex, and N-terminal pro-brain natriuretic peptide levels, the risk of the primary outcome was significantly higher in patients with a BMI below 23.5 (HR 1.27; 95% CI 1.04 to 1.56) and in those with a BMI of 35 kg/m^2^ or more (HR 1.27; 95% CI 1.06 to 1.52) compared to the reference group. A similar pattern was observed for both all-cause mortality and heart failure hospitalization.
Ather et al., 2011 [41]	Observational cohort study	2843 patients with HFpEF &&&and 6599 with HFrEF	≥50% and <50%	24 months	The lack of obesity was identified as an independent predictor of all-cause mortality in both HFpEF and HFrEF patients.
Powell-Wiley et al., 2018 [42]	Observational cohort study	39,647	≥50% and <50%	1 and 12 months	In individuals with HFpEF, higher BMI was associated with reduced 30-day mortality, showing a protective effect up to a BMI of 30 kg/m^2^. Above this level, the risk increased slightly (BMI of 30 kg/m^2^ versus 18.5 kg/m^2^, HR 0.63; 95% CI 0.54 to 0.73).
Iorio et al., 2018 [43]	Observational cohort study	2314 &&&(1373 with HFpEF)	≥50% and <50%	31 months	The absence of obesity was linked to higher overall mortality in both HFpEF and HFrEF groups.
Padwal et al., 2014 [44]	Meta-analysis of patient-level data from 14 heart failure dedicated studies	23,967	≥50% and <50%	36 months	In patients with chronic heart failure, the obesity paradox was observed in both HFrEF and HFpEF. Mortality in both heart failure subtypes followed a U-shaped curve, with the lowest mortality rates occurring at a BMI of 30.0–34.9 kg/m^2^.
Zhang et al., 2019 [45]	Dose-response meta-analysis of 10 studies	96,424 (59,263 with HFpEF)	≥50% and <50%	N/R	In patients with heart failure, the relationship between BMI and mortality follows a U-shaped curve, with the lowest risk observed at a BMI of 32–33 kg/m^2^ for both HFpEF and HFrEF.
Gentile et al., 2021 [46]	Observational cohort study	5155 (19% HFpEF)	≥50% and <50%	40 months	Mild obesity was independently linked to improved survival across the full range of LVEF. However, the survival advantage associated with obesity was preserved only in cases of non-ischemic heart failure.
Prausmüller et al., 2023 [47]	Large-scale cohort study	6744 individuals with HFpEF (25% with T2DM)	>50%	47 months	In the overall cohort, with a BMI of 22.5–24.9 kg/m^2^ serving as the reference, the unadjusted HR for all-cause mortality was elevated in patients with a BMI below 22.5 kg/m^2^ (HR 1.27; 95% CI 1.09–1.48) and decreased in those with a BMI of 25 kg/m^2^ or higher. After adjusting for multiple variables, BMI continued to show a significant inverse relationship with survival in patients without T2DM.

Abbreviations: BMI, body-mass index; HFpEF, heart failure with preserved ejection fraction; HFpEF, heart failure with reduced ejection fraction; CI, confidence interval; HR, hazard ratio; LVEF, left ventricular ejection fraction; N/R, not reported; RCT, randomized controlled trial; T2DM, type 2 diabetes mellitus.

**Table 2:** Effects of glucagon-like peptide-1 receptor agonists on epicardial adipose tissue.

Author	Inclusion Criteria	Sample Size	Follow-up Duration (Weeks)	GLP-1 Receptor Agonist	Imaging Modality	Change (%) in Epicardial Adipose Tissue Thickness
Iacobellis et al., 2017 [52]	T2DM and obesity	95	24	Liraglutide	Echocardiography	−42
Morano et al., 2015 [53]	T2DM	27	18	Liraglutide or exenatide	Echocardiography	−13
Iacobellis et al., 2020 [54]	T2DM and obesity	30	12	Semaglutide	Echocardiography	−20
		30	12	Dulaglutide	Echocardiography	−20
Lit et al., 2020 [55]	T2DM and obesity	21	12	Liraglutide	CMR	−29

Abbreviations: CMR, cardiac magnetic resonance, T2DM, type 2 diabetes mellitus.

**Table 3:** Studies evaluating the effect of glucagon-like peptide-1 receptor agonists on clinical outcomes in patients with obesity-related HFpEF.

Author/Study	Study Design	Participants	Intervention	Primary Endpoint	Outcomes
Kosiborod et al., 2023 [37]	Placebo-controlled, double-blind RCT	529 patients with HFpEF and BMI ≥ 30 without T2DM	Once weekly subcutaneously administered semaglutide (2.4 mg) or placebo for 52 weeks	Dual primary end points were the change from baseline in the KCCQ-CSS, and the change in body weight	In patients with HFpEF and obesity, treatment with semaglutide (2.4 mg) resulted in more pronounced reductions in symptoms and physical limitations, greater enhancements in exercise capacity, and more pronounced weight loss compared to placebo
Kosiborod et al., 2024 [94]	Placebo-controlled, double-blind RCT	616 patients with HFpEF, BMI ≥ 30 and T2DM	Once weekly subcutaneously administered semaglutide (2.4 mg) or placebo for 52 weeks	Dual primary end points were the change from baseline in the KCCQ-CSS, and the change in body weight	In patients with obesity-related HFpEF and T2DM, semaglutide led to more significant reductions in heart failure-related manifestations and physical limitations, as well as greater weight loss, compared to placebo over a 1-year period.
Butler et al., 2023 [95]	Prespecified analysis of the STEP-HFpEF RCT	529 patients with HFpEF and BMI ≥ 30 without T2DM	Once weekly subcutaneously administered semaglutide (2.4 mg) or placebo for 52 weeks	Dual primary end points were the change from baseline in the KCCQ-CSS, and the change in body weight	In patients with HFpEF and obesity, semaglutide 2.4 mg led to improvements in symptoms, physical limitations, and exercise capacity, while also reducing inflammation and body weight consistently across different LVEF categories (i) 45% to 49% (n = 85), (ii) 50% to 59% (n = 215), and (iii) ≥60% (n = 229))
Kosiborod et al., 2024 [96]	Prespecified analysis of the STEP-HFpEF RCT	529 patients with HFpEF and BMI ≥ 30 without T2DM	Once weekly subcutaneously administered semaglutide (2.4 mg) or placebo for 52 weeks	Dual primary end points were the change from baseline in the KCCQ-CSS, and the change in body weight based on KCCQ-CSS tertiles at baseline	In patients with HFpEF and obesity, semaglutide resulted in significant improvements in HF-related symptoms, physical limitations, exercise function, inflammation, body weight, and NT-proBNP levels, regardless of baseline health status
Borlaug et al., 2023 [97]	Prespecified analysis of the STEP-HFpEF RCT	529 patients with HFpEF and BMI ≥ 30 without T2DM	Once weekly subcutaneously administered semaglutide (2.4 mg) or placebo for 52 weeks	Change in the KCCQ-CSS and body weight across obesity classes I-III (BMI 30.0–34.9 kg m^2^, 35.0–39.9 kg m^2^ and ≥ 40 kg m^2^) and according to body weight loss with semaglutide after 52 weeks	In participants with the obesity phenotype of HFpEF, semaglutide enhanced symptoms, physical limitations, and exercise capacity, while also reducing inflammation and body weight across various obesity levels. Among those treated with semaglutide, the degree of benefit was directly proportional to the extent of weight loss achieved.
Butler et al., 2024 [98]	Pooled analysis of the STEP-HFpEF and STEP-HFpEF-DM trials	1145 patients with HFpEF and BMI ≥ 30 with or without T2DM	Once weekly subcutaneously administered semaglutide (2.4 mg) or placebo for 52 weeks	Change from baseline in the KCCQ-CSS, and the change in body weight	In this predetermined combined analysis of the STEP-HFpEF and STEP-HFpEF DM trials, semaglutide demonstrated superior efficacy over placebo in alleviating heart failure-related symptoms, reducing physical limitations, and decreasing body weight in subjects with obesity-related HFpEF. These benefits were largely consistent across various patient demographic and clinical characteristics. Moreover, semaglutide was well tolerated by the participants.
Schou et al., 2024 [99]	Prespecified analysis of pooled data from the the STEP-HFpEF and STEP-HFpEF-DM trials	1145 patients with HFpEF and BMI ≥ 30 with or without T2DM	Once weekly subcutaneously administered semaglutide (2.4 mg) or placebo for 52 weeks	Change in NYHA functional class from baseline to 52 weeks	In patients with obesity-related HFpEF, those treated with semaglutide experienced less deterioration and greater improvement in NYHA functional class compared to those receiving placebo at 52 weeks. Semaglutide consistently enhanced heart failure-related symptoms, physical limitations, and exercise capacity, while also reducing body weight and biomarkers of inflammation and congestion across all NYHA functional class categories. Notably, the improvements in health status were particularly pronounced in patients with NYHA functional classes III/IV.
Verma et al., 2024 [59]	Prespecified secondary analysis of pooled data from STEP-HFpEF and STEP-HFpEF DM	1145 patients with HFpEF and BMI ≥ 30 with or without T2DM	Once weekly subcutaneously administered semaglutide (2.4 mg) or placebo for 52 weeks	Change from baseline in the KCCQ-CSS, and the change in body weight were compared between sexes	In patients with obesity-related HFpEF, semaglutide 2.4 mg led to a more substantial reduction in body weight in women, while delivering comparable improvements in heart failure-related symptoms, physical limitations, and exercise function across both sexes
Petrie et al., 2024 [100]	Prespecified secondary analysis of pooled data from STEP-HFpEF and STEP-HFpEF DM	1145 patients with HFpEF and BMI ≥ 30 with or without T2DM	Once weekly subcutaneously administered semaglutide (2.4 mg) or placebo for 52 weeks	Change from baseline in the KCCQ-CSS, and the change in body weight were compared by baseline NT-proBNP	In individuals with obesity-related HFpEF, semaglutide led to reductions in NT-proBNP levels. Those with higher baseline NT-proBNP achieved comparable weight loss but experienced more substantial improvements in heart failure symptoms and physical limitations compared to those with lower NT-proBNP.
Shah et al., 2024 [101]	Pooled analysis of the STEP-HFpEF and STEP-HFpEF-DM trials	1145 patients with HFpEF and BMI ≥ 30 with or without T2DM	Once weekly subcutaneously administered semaglutide (2.4 mg) or placebo for 52 weeks	Change from baseline in the KCCQ-CSS, and the change in body weight across baseline diuretic use groups (no diuretics, non-loop diuretics only, and loop diuretics [< 40, 40, and > 40 mg/day furosemide equivalents]); and changes in loop diuretic use and dose over 52 weeks	In patients with obesity-related HFpEF, semaglutide significantly alleviated heart failure-related symptoms and physical limitations across different diuretic use subgroups, with particularly notable benefits observed in those using loop diuretics at baseline. The reductions in weight and enhancements in exercise function with semaglutide compared to placebo were uniform across all diuretic use categories. Additionally, semaglutide treatment was associated with a decrease in both the use and dosage of loop diuretics from baseline to 52 weeks.
Pérez-Belmonte et al., 2022 [102]	Real-world retrospective observational study	136 patients with T2DM, obesity, and heart failure	Once weekly subcutaneously administered semaglutide (maintenance dose 0.5 mg or 1.0 mg) for 12 months	Change from baseline in the KCCQ-CSS, New York Heart Association (NYHA) classification, and in NT-pro-BNP levels	In patients treated with semaglutide, there was a significantly greater improvement in the KCCQ total symptom score (59.0 ± 24.1 vs 79.9 ± 28.4 points, *p* < 0.01), a decrease in the percentage of patients with NYHA l class III (from 40.4% to 16.2%, *p* < 0.01), and a decrease in NT-pro-BNP levels compared to semaglutide non-users. Additionally, there was a reduction in emergency department visits, hospitalizations for heart failure, and all-cause hospitalizations in semaglutide group.
SUMMIT [103]	Randomized, double-blind, placebo-controlled Phase 3 trial	700 patients with NYHA Class II–IV and increased NT-proBNP, structural heart disease, or heart failure decompensation within 1 year	Tirzepatide administered subcutaneously vs. placebo	(i) All-cause mortality, heart failure events, 6MWD test, and KCCQ from baseline to week 120; &&&(ii) 6MWD test variation from baseline to week 52	(Not published yet)

Abbreviations: BMI, body-mass index; HFpEF, heart failure with preserved ejection fraction; KCCQ-CSS, Kansas City Cardiomyopathy Questionnaire clinical summary score; LVEF, left ventricular ejection fraction; RCT, randomized controlled trial; NT-pro-BNP, N-terminal pro-brain natriuretic peptide; T2DM, type 2 diabetes mellitus; 6MWD, 6 min walking distance.

## Data Availability

The data generated in this research will be shared upon reasonable request to the corresponding author.

## Abbreviations

6MWD, 6 min walk distance; BMI, body mass index; GIP, glucose-dependent insulinotropic polypeptide; GLP-1 RAs, glucagon-like peptide-1 receptor agonists; HF, heart failure; HFpEF, heart failure with preserved ejection fraction; HFrEF, reduced ejection fraction; KCCQ-CSS, Kansas City Cardiomyopathy Questionnaire clinical summary score; LVEF, left ventricular ejection fraction; MRAs, mineralocorticoid receptor antagonists; SGLT2, sodium–glucose cotransporter 2; T2DM, type 2 diabetes mellitus; VCAM, vascular cell adhesion molecule.
